# c-Ki-*ras* mutations in colorectal adenocarcinomas from a country with a rapidly changing colorectal cancer incidence

**DOI:** 10.1038/sj.bjc.6690683

**Published:** 1999-09

**Authors:** W-Y Tang, J Elnatan, Y-S Lee, H-S Goh, D R Smith

**Affiliations:** Molecular Biology Laboratory, Tan Tock Seng Hospital, Moulmein Road, Singapore, 308433, Republic of Singapore; Department of Pathology, National University of Singapore, National University Hospital, Lower Kent Ridge Road, Singapore, 119074, Republic of Singapore

**Keywords:** colon cancer, *ras*, proto-oncogene, mutation

## Abstract

We have examined the incidence of mutation of the c-Ki-*ras* proto-oncogene in colorectal adenocarcinomas from two different time periods, namely 1962–1966 and 1994–1996. The first cohort of samples consisted of formalin-fixed, archival paraffin block and represent the oldest colorectal cancer samples for which *ras* mutation has been examined, while the second cohort of tumours were fresh, flash-frozen samples representative of genetic events occurring in contemporary times. Analysis of mutation status was undertaken by a mismatch-specific oligonucleotide hybridization analysis of exon 1 of the c-Ki-*ras* proto-oncogene after amplification by the polymerase chain reaction. Mutations in codon 12 or 13 of c-Ki-*ras* were detected in 28% (14/50) of contemporary samples, a figure consistent with locally established mutation rates. In contrast no mutation was detected in any of the 18 samples from the earlier period, a result that is statistically significant (*P* = 0.007). Age-standardized rates of colorectal cancer in Singapore have seen a marked increase over the last 30 years, and for the first time we have shown that such an increase in colorectal cancer is associated, at least in part with an increase in incidence of a specific mutagenic change. © 1999 Cancer Research Campaign


*British Journal of Cancer* (1999) **81**(2), 237–241
© 1999 Cancer Research Campaign
Article no. bjoc.1999.0683

### c-Ki-*ras* mutations in colorectal adenocarcinomas from a country with a rapidly changing colorectal cancer incidence

**W-Y Tang[1], J Elnatan[1], Y-S Lee[2], H-S Goh[1] and DR Smith[1]**

[1]Molecular Biology Laboratory, Tan Tock Seng Hospital, Moulmein Road, Singapore 308433, Republic of Singapore; [2]Department of Pathology, National University of Singapore, National University Hospital, Lower Kent Ridge Road, Singapore 119074, Republic of Singapore

**Summary** We have examined the incidence of mutation of the c-Ki-*ras* proto-oncogene in colorectal adenocarcinomas from two different time periods, namely 1962–1966 and 1994–1996. The first cohort of samples consisted of formalin-fixed, archival paraffin block and represent the oldest colorectal cancer samples for which *ras* mutation has been examined, while the second cohort of tumours were fresh, flash-frozen samples representative of genetic events occurring in contemporary times. Analysis of mutation status was undertaken by a mismatch-specific oligonucleotide hybridization analysis of exon 1 of the c-Ki-*ras* proto-oncogene after amplification by the polymerase chain reaction. Mutations in codon 12 or 13 of c-Ki-*ras* were detected in 28% (14/50) of contemporary samples, a figure consistent with locally established mutation rates. In contrast no mutation was detected in any of the 18 samples from the earlier period, a result that is statistically significant ($P$ = 0.007). Age-standardized rates of colorectal cancer in Singapore have seen a marked increase over the last 30 years, and for the first time we have shown that such an increase in colorectal cancer is associated, at least in part with an increase in incidence of a specific mutagenic change.

© 1999 Cancer Research Campaign

**Keywords:** colon cancer; *ras*; proto-oncogene; mutation

Singapore is a small city state located approximately 1° above the equator. The population of approximately 3 million citizens is comprised of three main ethnic groups; the Chinese who comprise some 77% of the population, the Malays, who comprise some 14% of the population and the Indians who comprise approximately 7% of the population. Each of these races has significantly different age-standardized rates of colorectal cancer (see Table 1), with the Chinese citizens having a 2–2.5-fold higher risk of getting colorectal cancer than their Indian or Malay neighbours (Chia et al, 1996).

The incidence of colorectal cancer throughout the population has been rising steadily. In 1972, the end of the first 5-year period in which comprehensive national cancer records were kept, the age-standardized rates of colorectal cancer were 19.9 per 100 000 for males and 15.6 per 100 000 for females. Colorectal cancer was the fourth most common cancer in males, and the fifth most common cancer in females. Today the situation is markedly different, and the records for the period 1988–1992, the latest for which records have been published, colorectal cancer incidence is 36.4 and 28.5 per 100 000 for males and females respectively, and ranks as the second most common cancer for both males and females (Chia et al, 1996). As such, Singapore offers unique opportunities to study the molecular events in tumorigenesis, both with respect to a single population that is undergoing a rapid increase in incidence of colorectal cancer, as well as by comparisons between population groups who essentially experience the same social and living conditions, but have markedly different rates of colorectal cancer.

*Received 7 October 1998*
*Revised 8 February 1999*
*Accepted 11 March 1999*

*Correspondence to:* DR Smith

Colorectal cancer is believed to arise by the accumulation of genetic damage, in a process that may take years, or potentially decades (Fearon and Vogelstein, 1990; Fearon and Jones, 1992). Many of these changes have been well documented, and can include activation of oncogenes as well as the inactivation of tumour suppressor genes and genes of the mismatch repair family of genes (Baba, 1997).

Approximately 40% of colorectal adenocarcinomas have been shown to contain activated copies of the c-Ki-*ras* proto-oncogene (Breivik et al, 1994; Kiaris and Spandidos, 1995; Elnatan et al, 1996) This gene belongs to the *ras* family of proto-oncogenes, three closely related genes that code for proteins 188 or 189 amino acids long (Barbacid, 1987; Bos, 1989; Grand and Owen, 1991), which exhïbit intrinsic GTPase activity (Grand and Owen, 1991). All members of the *ras* family are converted to active oncogenes in naturally occurring tumours by point mutations in codons 12, 13 or 61 (Barbacid, 1987), although there is a strong preference for mutations in codons 12 or 13 (Bos, 1989; Yanez et al, 1987). These mutations abolish GTPase activity, and result in constitutive stimulation of autonomous growth and contribute to neoplastic

**Table 1** Age-standardized rates of colorectal cancer in Singapore

| | 1968–1972 | | 1988–1992 | |
|---|---|---|---|---|
| | Males | Females | Males | Females |
| Chinese | 22.2 | 16.6 | 42.2 | 31.2 |
| Malay | 8.6 | 6.0 | 19.1 | 14.3 |
| Indian | 10.7 | 9.0 | 13.6 | 11.3 |
| Population | 19.9 | 15.6 | 36.4 | 28.5 |

Age-standardised rates for colorectal cancer by sex and race for time periods 1968–1972 and 1988–1992 (adapted from Chia et al, 1996)

development (Barbacid, 1987; Bos, 1989; Grand and Owen, 1991).

The particular member of the *ras* gene family that is found to be activated is characteristic of a particular tumour type, and in colorectal adenocarcinomas the majority of *ras* activation is associated with c-Ki-*ras* (Bos et al, 1987). Activation of the c-Ki-*ras* gene has been found to be associated with a number of factors such as patient age, sex and tumour location (Breivik et al, 1994; Elnatan et al, 1996), and the presence of mutations has been variously correlated with neoplastic progression (Vogelstein et al, 1988; Fearon and Vogelstein, 1990; Fearon and Jones, 1992), pattern of disease dissemination (Finkelstein et al, 1993*a*, 1993*b*) and patient survival (Elnatan et al, 1996; Smith et al, 1996).

We were interested to discover whether the rate of c-Ki-*ras* mutation was constant in a single population that had experienced colorectal cancer as both a high incidence and a low incidence disease within the span of a single generation, and to possibly determine whether the spectrum of mutations in the c-Ki-*ras* gene had undergone significant alterations. To undertake this we analysed tumours harvested some 30–35 years ago in parallel with tumours collected from contemporary patients. Results showed that the mutation rates are significantly different between the two cohorts, and for the first time offering a potential link between the increase in the incidence of colorectal cancer seen in Singapore and a specific genetic change.

### MATERIALS AND METHODS

### Patients and tumours

Tumours analysed were from patients undergoing colonic resection for colorectal adenocarcinoma either during the period 1962–1966 or during the period 1994–1996. Tumours from the contemporary period were collected along with matched normal mucosa from the same patient and stored at –80°C until analysed. Tumours from the period 1962–1966 were obtained as several contiguous 10-μm sections from paraffin blocks histologically verified to contain colorectal adenocarcinoma.

### DNA preparation, PCR and DNA sequencing

Genomic DNA was extracted from tumour samples containing at least 70% neoplastic cells, as assessed histochemically, along with genomic DNA from matched normal mucosa from the same patient as described previously (Khine et al, 1994). Genomic DNA was prepared from archival paraffin sections using the Prep-A-Gene (BioRad, Hercules, CA, USA) protocol of Wang et al (1994). A 176-bp fragment of exon 1 of the c-Ki-*ras* gene was amplified from all DNA samples using custom synthesized oligonucleotides 5′-CATGTTCTAATATAGTCACA-3′ (forward); and 5′-AACAAGAATTACCTCTATTG-3′ (reverse) essentially as described previously (Elnatan et al, 1996; Smith et al, 1996). Polymerase chain reactions (PCR) were as previously described (Elnatan et al, 1996; Smith et al, 1996), with the modification that to equalize yields of amplicon from the two source of starting material, archival samples were subjected to 40 cycles while fresh, flash-frozen materials (tumour and mucosa) were subjected to 30 cycles.

For DNA sequencing the PCR products were purified by agarose gel electrophoresis and excised from the gel matrix after which templates were purified using the Qiaex PCR Purification Kit (Qiagen, Hilden, Germany). DNA sequencing reactions were performed using the Big Dye Termination reaction kit (PE Applied Biosystems, Foster City, CA, USA) on approximately 60–90 ng of template from above. Analysis was undertaken on an automated ABI 377-18 DNA Sequencer (PE Applied Biosystems).

### Mismatch-specific oligonucleotide hybridization

Mismatch-specific oligonucleotide hybridization was performed essentially as described by Verlaan de Vries et al (1986) with some modifications as described previously (Elnatan et al, 1996; Smith et al, 1996) using a panel of primers specific for all possible mutations of the c-Ki-*ras* proto-oncogene in codons 12 and 13 (Human *ras* Muta-Lyzer Probe Panels, Clontech Laboratories Inc., Palo Alto, CA, USA).

### RESULTS

A total of 68 colorectal adenocarcinomas were examined for mutation of the c-Ki-*ras* proto-oncogene. Eighteen of these samples were archival paraffin sections obtained from formalin-treated, paraffin-embedded blocks of tumour biopsy samples collected from patients operated on between 1962 and 1966. The remaining 50 samples were from patients operated on during the period 1992–1994 and kept as fresh, flash-frozen specimens, along with their matched normal mucosa until required for analysis. No statistically significant difference was found between the two cohorts with respect to patient age, sex, race, tumour location and stage (Table 2). All tumour samples were histologically confirmed to contain at least 70% tumour cells.

Genomic DNA was extracted from contemporary tumour samples in parallel with genomic DNA from matched normal mucosa of the same patient by standard methodologies (Khine et al, 1994). Several methodologies were attempted for extraction of genomic DNA by archival samples, with consistent results being

**Table 2** Clinical data for archival (1962–1966) and contemporary (1994–1996) colorectal adenocarcinoma samples

| | Contemporary samples | Archival samples | Significance |
|---|---|---|---|
| Sex | | | |
| Male | 33 | 13 | |
| Female | 17 | 5 | NS ($P$ = 0.62) |
| Age | | | |
| Mean (±SD) | 62.4 ± 14.9 | 56.5 ± 11.6 | NS ($P$ = 0.13) |
| Range | 25–91 | 35–76 | |
| Location | | | |
| Proximal | 16 | 5 | |
| Distal | 34 | 13 | NS ($P$ = 0.73) |
| Stage | | | |
| A | 7 | 2 | |
| B | 15 | 10 | NS ($P$ = 0.14) |
| C | 28 | 6 | |
| Race | | | |
| Chinese | 44 | 15 | NS ($P$ = 0.61) |
| Others | 6 | 3 | |

The two cohorts are not significantly different in composition with respect to sex (Fisher's exact test), age (ANOVA), tumour location (Fisher's exact test), race (Fisher's exact test) and stage ($\chi^2$ test). Tumours are staged as (**A**) tumour within intestinal wall; (**B**) tumour extends beyond serosa, no lymph node involvement and (**C**) lymph node metastasis. NS, not significant.

© 1999 Cancer Research Campaign

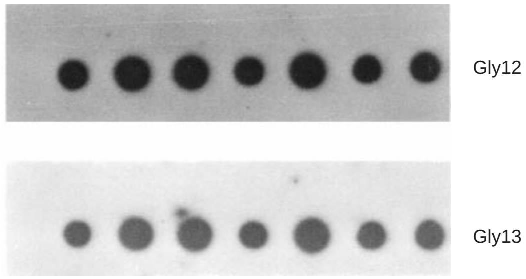

**Figure 1** Representative c-Ki-*ras* mismatch-specific oligonucleotide hybridization analysis of seven archival (> 30 years) colorectal adenocarcinoma specimens. Shown here are results for probes for Gly12 and Gly13 (wild-type sequences). All other probes were negative

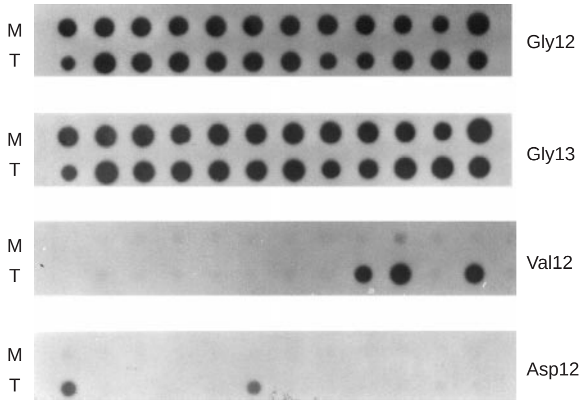

**Figure 2** Representative c-Ki-*ras* mismatch-specific oligonucleotide hybridization analysis of 12 contemporary colorectal adenocarcinoma specimens (bottom row: T) together with their normal matched mucosa harvested from the same patient (top row: M). Results are shown for Gly12 and Gly13 (wild-type sequences), as well as for Val12 and Asp12 (mutated codons). All other probes were negative in these samples obtained by the Prep-A-Gene methodology of Wang et al (1994; data not shown). Exon 1 of the c-Ki-*ras* gene was amplified by PCR, and samples dotted onto nylon support membranes. All samples were analysed for mutations occurring in codon 12 and 13 of c-Ki-*ras* by mismatch-specific oligonucleotide hybridization (Verlaan de Vries et al, 1986). Control hybridizations for both codon 12 and 13 wild-type sequences were also carried out (Figures 1 and 2).

Sixteen mutants were detected in 14 samples (two tumours contained two mutant codons) from the contemporary cohort. Hence 28% of samples were mutated (14/50) and 8% (16/200) of codons. Ninety-three per cent (15/16) of the mutations occurred in codon 12 and 7% (1/16) in codon 13 (Figure 2). Both the frequency of the c-Ki-*ras* mutation and the distribution of mutants (Table 3) were consistent with our previously published results on c-Ki-*ras* gene mutations in the local Singaporean population (Elnatan et al, 1996).

In contrast, archival samples from 1962 to 1966 showed no evidence of mutation in any sample, a rate statistically different from that found in the contemporary cohort both with respect to the number of tumours bearing *ras* mutations (Samples 1992–1994: 28% [14/50]; Samples 1962–1966 0% [0/18]; $P$ = 0.007, Fisher's exact test) and to the number of codons (codons 1992–1994: 8% [16/200]; codons 1962–1996 0% [0/72]; $P$ = 0.008, Fisher's exact test). Probes for wild-type codon 12 and 13 both gave normal level signals (Figure 2). To further confirm the identity of the PCR product from the archival samples, samples were also analysed by DNA sequencing using an automated DNA sequencer. Sequencing analysis confirmed the identity of the product as being exon 1 of the c-Ki-*ras* gene (Figure 3).

**Table 3** Distribution of c-Ki-*ras* mutants

| | Established local rates* | Present cohort (contemporary) | Present cohort (archival) |
|---|---|---|---|
| Asp12 | 7% (14/210) | 14% (7/50) | 0% (0/18) |
| Val 12 | 8% (17/210) | 10% (5/50) | 0% (0/18) |
| Cys12 | 5% (10/210) | 2% (1/50) | 0% (0/18) |
| Ser12 | 2% (4/210) | 2% (1/50) | 0% (0/18) |
| Ala12 | 1.5% (3/210) | 2% (1/50) | 0% (0/18) |
| Asp13 | 5% (11/210) | 2% (1/50) | 0% (0/18) |
| Cys13 | 1.5% (3/210) | 0% (0/50) | 0% (0/18) |
| Total | 30% (62/210) | 32% (16/50) | 0% (0/18) |

The specific c-Ki-*ras* mutants in the present cohorts are listed, alongside previously established c-Ki-*ras* data for colorectal adenocarcinomas from Singapore (*Elnatan et al, 1996).

### DISCUSSION

In this study we have reported a statistically different rate of c-Ki-*ras* mutation in samples of colorectal adenocarcinomas harvested 30–35 years ago to the rate of mutation detected in contemporary samples. Indeed, we were unable to detect any cases of c-Ki-*ras* mutation in the archival samples. This result was somewhat surprising. The c-Ki-*ras* proto-oncogene is currently found mutated in 20–40% of colorectal adenocarcinomas, dependent upon factors including the age and sex of the patient as well as the location of the tumour (Breivik et al, 1994; Elnatan et al, 1996), and c-Ki-*ras* activation is believed to play a significant role in tumorigenesis, possibly being involved in the transition from adenoma to carcinoma (Fearon and Vogelstein, 1990; Fearon and Jones, 1992). Our own data have shown a significant association between c-Ki-*ras* activation and tumour progression in tumours originating on the left of the colorectum and an association with a poorer prognosis (Elnatan et al, 1996).

However, recently several lines of evidence have suggested that *ras* activation may not be causally involved in tumour progression. In particular the findings that *ras* activation can be detected in phenotypically normal tissues (Keohavong et al, 1997; Zhu et al, 1997), and in small non-dysplastic lesions of low tumorigenic potential (Jen et al, 1994) as well as the observation that activated *ras* genes are present in tumour cells at levels approximately 100-fold less than that required to induce transformation in cell lines (Hua et al, 1997). These findings, coupled with those that show that *ras* activation is actually higher in aberrant crypt foci (Otori et al, 1995; Yamashita et al, 1995*a*, 1995*b*) – the purported precursor to adenomas – than in developed adenocarcinomas suggest that *ras* activation may simply reflect the mutational burden of a tumour, rather that being specifically involved in the tumorigenic process.

We have previously shown that both p53 point mutation (Goh et al, 1995) and c-Ki-*ras* point mutation (Elnatan et al, 1996) are significantly associated with a poorer patient prognosis in tumours originating in tumours on the left of the colorectum. Perhaps more

*© 1999 Cancer Research Campaign*

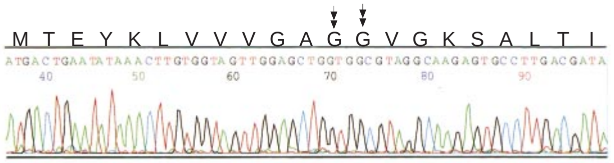

**Figure 3** Representative DNA electropherogram of a portion of the c-Ki-*ras* gene from an archival (> 30 years) specimen. Nucleotides encoding the first 21 amino acids are depicted, along with the amino acid sequence in single letter code. Codons Gly12 and Gly13 are indicated by arrowheads interestingly we have also shown that when considering these two changes together the effect on patient prognosis is additive with patients showing both p53 and *ras* mutation showing a poorer prognosis than patients showing either one of these changes, and these patients in turn showing a poorer prognosis than patients who evinced neither of these changes (Smith et al, 1996). In a Cox regression analysis we noted that the terms for both p53 mutation and c-Ki-*ras* mutation were excluded in favour of a term that simply described the number of changes seen in a tumour (0, 1 or 2). As such, this analysis would seem to indicate that a better idea of patient prognosis could be determined by obtaining some idea of mutational burden.

The cause of mutations in the c-Ki-*ras* proto-oncogene remain unclear, although some authors have suggested that bile acids may be implicated in the tumorigenic process (Cohen et al, 1989; Chaplin, 1998). Other studies have shown that cooked-meat heterocyclic amines such as 2-amino-3-methylimidazo[4,5-f]quinoline can induce aberrant crypt foci with a high rate of *ras* mutation in animals (Tachino et al, 1995). The observation that the rate of mutation in tumours originating on the right of the colorectum is significantly higher than that found in tumours originating on the left of the colorectum while the spectrum of mutations detected is similar (Elnatan et al, 1996) is certainly consistent with a decreasing gradient of mutagen along the length of the human colorectum. As such c-Ki-*ras* activation is potentially a 'fingerprint' marker of exogenous mutagens.

Many studies have tried to elucidate a relationship between colorectal cancer and diet and despite the difficulties of these studies a common consensus would suggest a possible relationship between consumption of certain foodstuffs and an increased susceptibility to, or protection from colorectal cancer (World Cancer Research Fund, 1997). In particular, several studies have indicated a protective role for cruciferous vegetables such as cabbage and broccoli (Graham et al, 1978; Haenszel et al, 1980; Colditz et al, 1985), while other studies have implicated an increased risk of developing colorectal cancer based on either high meat consumption (Haenszel et al, 1973; La Vecchia et al, 1988) or a high meat to vegetable ratio (Manousos et al, 1983; Lee et al, 1989). In Singapore, while there are several studies on recent food consumption trends (Gourley et al, 1988; Lee et al, 1989), data on diet 30 years ago are somewhat more scanty. However, what data there are (Lee and Gourley, 1987) clearly point to a significant increase in the consumption of meat/offal and oily foods over the last 30 years. As such the data presented here indicate a somewhat tenuous link between changing diet, increasing incidence of colorectal cancer and increasing incidence of c-Ki-*ras* mutation suggesting that further studies using old (> 30 years) paraffin blocks may provide valuable insights into the tumorigenic process.

### ACKNOWLEDGEMENTS

This work was supported by grant number RSCH/97/007 from Tan Tock Seng Hospital.

### REFERENCES

Baba S (1997) Recent advances in molecular genetics of colorectal cancer. *World J Surg* **21**: 678–687

Barbacid M (1987) *ras* genes. *Annu Rev Biochem* **56**: 779–827

Bos JL, Fearon ER, Hamilton SR, Verlaan-de Vries M, van Boom JH, van der Eb AJ and Vogelstein B (1987) Prevalence of *ras* gene mutations in human colorectal cancers. *Nature* **327**: 293–297

Bos JL (1989) *ras* oncogenes in human cancer: a review. *Cancer Res* **49**: 4682–4689

Breivik J, Meling GI, Spurkland A, Rognum TO and Gaudernack G (1994) K-*ras* mutation in colorectal cancer: relations to patient age, sex and tumour location. *Br J Cancer* **69**: 367–371

Chaplin MF (1998) Bile acids, fibre and colon cancer: the story unfolds. *J Roy Soc Health* **118**: 53–61

Chia KS, Lee HP, Seow A and Shanmugaratnam K (1996) *Trends in Cancer Incidence in Singapore 1968–1992*. Singapore Cancer Registry Report No. 4.

Cohen AM, Shank B and Friedman MA (1989) Colorectal cancer. In: *Cancer: Principles and Practice of Oncology*, Devita VT, Hellman S and Rosenberg SA (eds), pp. 895–952. JB Lippincott: Philadelphia

Colditz GA, Branch LG, Lipnick RJ, Willett WC, Rosner B, Posner BM and Hennekens CH (1985) Increased green and yellow vegetable intake and lowered cancer deaths in an elderly population. *Am J Clin Nutr* **41**: 32–36

Elnatan J, Goh H-S and Smith DR (1996) C-Ki-*ras* activation and the biological behaviour of proximal and distal colonic adenocarcinomas. *Eur J Cancer* **32**: 491–497

Fearon ER and Jones PA (1992) Progressing toward a molecular description of colorectal cancer development. *FASEB J* **6**: 2783–2790

Fearon ER and Vogelstein B (1990) A genetic model for colorectal tumorigenesis. *Cell* **61**: 759–767.

Finkelstein SD, Sayegh R, Christensen S and Swalsky PA (1993*a*) Genotypic classification of colorectal adenocarcinoma. Biologic behavior correlates with K-*ras*-2 mutation type. *Cancer* **71**: 3827–3838

Finkelstein SD, Sayegh R, Bakker A and Swalsky P (1993*b*) Determination of tumor aggressiveness in colorectal cancer by K-*ras*-2 analysis. *Arch Surg* **128**: 526–531

Goh H-S, Yao J and Smith DR (1995) p53 point mutation and survival in colorectal cancer patients. *Cancer Res* **55**: 5217–5221

Gourley L, Duffy SW, Lee HP, Walker AM and Day NE (1988) A survey of household food purchases and dietary habits in relation to affluence among Singapore Chinese. *Eur J Clin Nutr* **42**: 333–343

Graham S, Dayal H, Swanson M, Mittelman A and Wilkinson G (1978) Diet in the epidemiology of cancer of the colon and rectum. *J Natl Cancer Inst* **61**: 709–714

Grand RJ and Owen D (1991) The biochemistry of *ras* p21. *Biochem J* **279**: 609–631

Haenszel W, Berg JW, Segi M, Kurihara M and Locke FB (1973) Large-bowel cancer in Hawaiian Japanese. *J Natl Cancer Inst* **51**: 1765–1779

Haenszel W, Locke FB and Segi M (1980) A case-control study of large bowel cancer in Japan. *J Natl Cancer Inst* **64**: 17–22

Hua VY, Wang WK and Duesberg PH (1997) Dominant transformation by mutated human *ras* genes *in vitro* requires more than 100 times higher expression than is observed in cancers. *Proc Natl Acad Sci USA* **94**: 9614–9619

Jen J, Powell SM, Papadopoulos N, Smith KJ, Hamilton SR, Vogelstein B and Kinzler KW (1994) Molecular determinants of dysplasia in colorectal lesions. *Cancer Res* **54**: 5523–5526

Keohavong P, Zhu D, Whiteside TL, Swalsky P, Bakker A, Elder EM, Siegfried JM, Srivastava S and Finkelstein SD (1997) Detection of infrequent and multiple K-*ras* mutations in human tumors and tumor-adjacent tissues. *Anal Biochem* **247**: 394–403

Khine K, Smith DR and Goh HS (1994) High frequency of allelic deletion on chromosome 17p in advanced colorectal cancer. *Cancer* **73**: 28–35

Kiaris H and Spandidos DA (1995) Mutations of *ras* genes in human tumours. *Int J Oncol* **7**: 413–421

La Vecchia C, Negri E, Decarli A, D'Avanzo B, Gallotti L, Gentile A and Franceschi S (1988) A case-control study of diet and colo-rectal cancer in northern Italy. *Int J Cancer* **41**: 492–498

*© 1999 Cancer Research Campaign*

Lee HP and Gourley L (1987) Fat and fibre in the Singapore diet. *Ann Acad Med Singapore* **16**: 408–411

Lee HP, Gourley L, Duffy SW, Esteve J, Lee J and Day NE (1989) Colorectal cancer and diet in an Asian population – a case-control study among Singapore Chinese. *Int J Cancer* **43**: 1007–1016

Manousos O, Day NE, Trichopoulos D, Gerovassilis F, Tzonou A and Polychronopoulou A (1983) Diet and colorectal cancer: a case-control study in Greece. *Int J Cancer* **32**: 1–5

Otori K, Sugiyama K, Hasebe T, Fukushima S and Esumi H (1995) Emergence of adenomatous aberrant crypt foci (ACF) from hyperplastic ACF with concomitant increase in cell proliferation. *Cancer Res* **55**: 4743–4746

Smith DR, Elnatan J, Yao J and Goh H-S (1996) Point mutation of the c-Ki-*ras* proto-oncogene and the p53 tumour suppressor gene in distal colonic adenocarcinomas. *Int J Oncol* **8**: 1165–1169

Tachino N, Hayashi R, Liew C, Bailey G and Dashwood R (1995) Evidence for *ras* gene mutation in 2-amino-3-methylimidazo [4,5-f]quinoline-induced colonic aberrant crypts in the rat. *Mol Carcinogen* **12**: 187–192

Verlaan-de Vries M, Bogaard ME, van den Elst H, van Boom JH, van der Eb AJ and Bos JL (1986) A dot-blot screening procedure for mutated *ras* oncogenes using synthetic oligodeoxynucleotides. *Gene* **50**: 313–320

Vogelstein B, Fearon ER, Hamilton SR, Kern SE, Preisinger AC, Leppert M, Nakamura Y, White R, Smits AM and Bos JL (1988) Genetic alterations during colorectal-tumor development. *N Engl J Med* **319**: 525–532

Wang W, Kumar P, Schwarz M, Malone G, Haworth A and Kumar S (1994) PCR amplification of 40 year old paraffin-embedded tumour tissues: comparison of four different DNA extraction and purification methods. *Int J Oncol* **5**: 453–457

World Cancer Research Fund (1997) Colon, rectum. In: *Food, Nutrition and the Prevention of Cancer: a Global Perspective*, pp. 216–251. American Institute of Cancer Research

Yamashita N, Minamoto T, Ochiai A, Onda M and Esumi H (1995*a*) Frequent and characteristic K-*ras* activation in aberrant crypt foci of colon. Is there preference among K-*ras* mutants for malignant progression? *Cancer* **75**: 1527–1533

Yamashita N, Minamoto T, Ochiai A, Onda M and Esumi H (1995*b*) Frequent and characteristic K-*ras* activation and absence of p53 protein accumulation in aberrant crypt foci of the colon. *Gastroenterology* **108**: 434–440

Yanez L, Groffen J and Valenzuela DM (1987) c-K-*ras* mutations in human carcinomas occur preferentially in codon 12. *Oncogene* **1**: 315–318

Zhu D, Keohavong P, Finkelstein SD, Swalsky P, Bakker A, Weissfeld J, Srivastava S and Whiteside TL (1997) K-*ras* gene mutations in normal colorectal tissues from K-*ras* mutation-positive colorectal cancer patients. *Cancer Res* **57**: 2485–2492

*© 1999 Cancer Research Campaign*
